# Recent advances in Norrish–Yang cyclization and dicarbonyl photoredox reactions for natural product synthesis

**DOI:** 10.3762/bjoc.21.177

**Published:** 2025-10-30

**Authors:** Peng-Xi Luo, Jin-Xuan Yang, Shao-Min Fu, Bo Liu

**Affiliations:** 1 College of Chemistry, Sichuan University, 29 Wangjiang Rd., Chengdu, Sichuan 610064, China

**Keywords:** dicarbonyls, natural product, Norrish–Yang cyclization, photoredox, total synthesis

## Abstract

In recent years, the Norrish–Yang cyclization and related photoredox reactions of dicarbonyls have been extensively utilized in natural product synthesis. This review summarizes the latest advancements in these reactions for constructing terpenoids, alkaloids, and antibiotics. Through Norrish–Yang cyclization, dicarbonyls (e.g., 1,2-diketones and α-keto amides) can efficiently construct sterically hindered ring structures, which can further undergo ring-opening or rearrangement reaction to assemble complex molecular frameworks. Additionally, quinone photoredox reactions involving single-electron transfer (SET) processes provide novel strategies for the stereoselective synthesis of useful structures such as spiroketals. This review, drawing on examples from recently reported natural product syntheses, elaborates on reaction mechanisms, factors governing regioselectivity and stereoselectivity, and the impact of substrate structures on reaction pathways. These reactions not only serve as robust tools for the streamlined synthesis of natural products but also establish a solid foundation for subsequent pharmaceutical investigations.

## Introduction

**Scheme 1:**
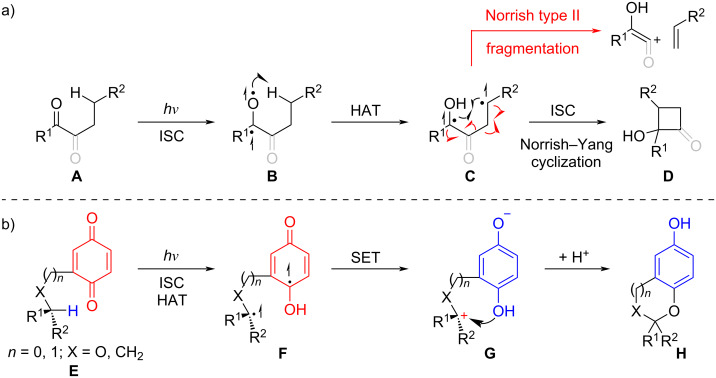
a) The mechanism of Norrish type II reaction and Norrish–Yang cyclization; b) The mechanism of the dicarbonyls photoredox reaction.

In the 1930s, Norrish documented the photodecomposition of aldehydes and ketones [1–3]. These studies revealed distinct reaction types, with the Norrish type II reaction being one of the most extensively characterized. The mechanism underlying the Norrish type II reaction proceeds via the following steps (Scheme 1a): photoexcitation of carbonyl compound **A** generates an excited singlet state, which undergoes intersystem crossing (ISC) to form the excited triplet state **B**. An intramolecular 1,5-hydrogen atom transfer (HAT) then ensues, producing the 1,4-diradical **C**, which can be converted into diverse products such as alkenes and enols (Scheme 1a). Notably, the 1,4-diradical intermediate is also capable of cyclization through radical coupling to form cyclobutanol **D**, a process systematically expanded upon by Yang's group at the University of Chicago [4], which later became known as the Norrish–Yang cyclization. In recent years, dicarbonyls, specifically 1,2-diketones, α-keto esters, α-keto amides, 1,4-quinones, and 1,2-quinones in this context, have emerged as versatile substrates in Norrish–Yang cyclizations, finding widespread application in natural products synthesis. Compared to monoketones, dicarbonyls (e.g., 1,2-diketones) offer distinct advantages: (1) they generally favor Norrish–Yang cyclization over the competing unproductive Norrish type II fragmentation pathway; (2) the long excitation wavelength of 1,2-diketones (λ_max_ ≈ 450 nm) enables their selective activation in the presence of other photochemically excitable groups [5–6]. Furthermore, the resulting α-hydroxy-β-lactams or 2-hydroxycyclobutanones can function either as inherent structural motifs in target natural products or as strained reactive intermediates, facilitating C–H functionalization via four-membered ring opening [6–11]. Quinones display distinct photochemical reactivities among dicarbonyl compounds, as they not only undergo Norrish–Yang cyclization but also engage in photorearrangement, photoreduction, and photoredox cyclization reactions [12–16]. The last of these, first reported in 1965 [17], has been the subject of extensive studies by Suzuki’s group [18]. In contrast to the direct radical coupling in Norrish–Yang cyclization, the distal biradical **F**, formed from quinone **E** through a pathway analogous to that of **C** in the photoredox process, subsequently undergoes intramolecular SET to generate a zwitterion **G**. This intermediate is then trapped by the proximal phenol, yielding the cyclization product **H** (Scheme 1b). Notably, the quinone is reduced, while the proximal C–H bond is subject to oxidation. Building on these mechanistic insights and the synthetic merits of dicarbonyls, Norrish–Yang cyclization and related photoredox reactions have been serving as powerful tools for constructing complex molecular architectures. Centered on the structural diversity and synthetic relevance of target natural products, this review is divided into three main sections: terpenoids, alkaloids, and antibiotics. Each section highlights the recent application of Norrish–Yang cyclization and related photoredox reactions in their total synthesis or model studies. Earlier literature pertaining to Norrish type I and II reactions in natural product synthesis has been comprehensively reviewed by Majhi [19] and thus lies beyond the scope of this article.

## Review

### 1 Terpenoids

#### 1.1 (+)-Cyclobutastellettolide B

**Scheme 2:**
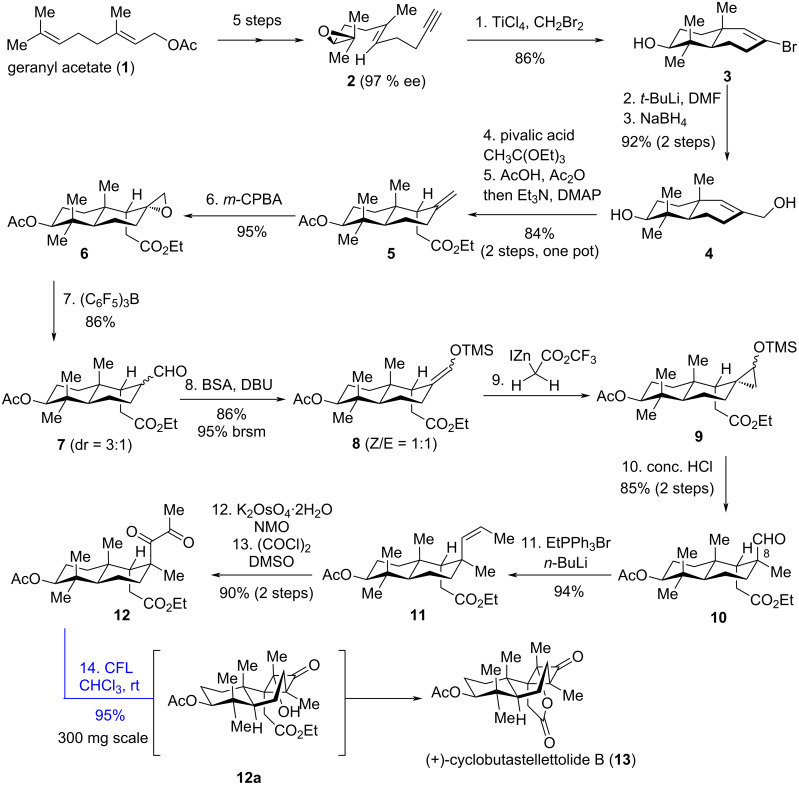
Total synthesis of (+)-cyclobutastellettolide B.

(+)-Cyclobutastellettolide B (**13**), featuring an unusual 6/6/4-fused tricyclic core with six stereocenters – including three contiguous quaternary stereocenters – was first isolated by Stonik et al. in 2019 from a *Stelletta sp*. sponge collected in Vietnamese waters [20]. This compound significantly elevates reactive oxygen species levels in murine peritoneal macrophages (73 ± 12%) and exhibits potential as a lead for developing immunomodulatory agents. In 2021, Yang’s group reported a concise enantioselective total synthesis of (+)-cyclobutastellettolide B, employing a late-stage biomimetic Norrish−Yang cyclization to construct the highly compact cyclobutanone motif [21]. The task commenced with epoxide **2** (Scheme 2), which was accessible via a known five-step protocol from geranyl acetate (**1**). Compound **2** was advanced to **3** using a tandem cyclization developed by the Yang group [22]. Installation of the hydroxymethyl group in **4** was achieved through sequential formylation and reduction. Compound **4** then underwent a one-pot, substrate-controlled diastereoselective Johnson−Claisen rearrangement/acetylation to install ester **5**. Treating **5** with *m*-CPBA (*meta*-chloroperoxybenzoic acid) induced epoxidation, which was then followed by a Meinwald rearrangement to accomplish aldehyde **7**. From **7**, a sequence involving silyl enol ether formation, Simmons−Smith cyclopropanation, and acid-mediated regioselective ring-opening installed the C8 quaternary methyl group in **10**. Subsequent transformation of **10** via sequential Wittig reaction, dihydroxylation, and Swern oxidation generated 1,2-diketone **12**, thus setting the stage for the Norrish–Yang reaction.

**Scheme 3:**
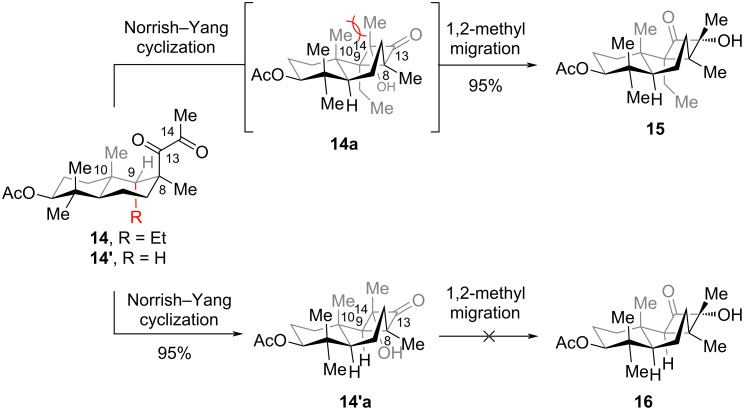
Norrish–Yang cyclization and 1,2-methyl migration.

Finally, irradiation of **12** with a compact fluorescent lamp (CFL) completed (+)-cyclobutastellettolide B (**13**) as the sole product in 95% yield. This transformation proceeded via sequential regio- and stereoselective Norrish−Yang annulation, followed by intramolecular lactonization mediated by **12a**. Notably, when the ethyl ester in **12** was replaced with a methyl group to form **14** with R = Et (Scheme 3), photoreaction of **14** led to **15** in 95% yield. This product arises from a Norrish–Yang cyclization/1,2-methyl migration cascade of **14** via intermediate **14a**. Intriguingly, the substrate with R = H (**14’**) underwent only Norrish–Yang cyclization (95% yield) without 1,2-methyl migration [23]. It is hypothesized that the substituent at C9 in compound **14** drives this migration, due to the highly sterically congested environment – exacerbated by the 1,3-strain between the methyl groups at C10 and C14. The observed 1,2-methyl migration also underscores the critical role of the preinstalled ester group in **12**: it suppresses potential methyl migration, enabling the total synthesis of **13** via in situ intramolecular lactonization as a key step.

Computational and experimental studies reveal that the regio- and stereoselectivity of the Norrish−Yang cyclization are governed by the influence of the methyl group at C10: (1) In terms of regioselectivity, the 1,3-strain between the methyl group at C10 and the substituent at C8 restricts the free rotation of the C8–C13 bond (although this steric hindrance can be circumvented through chair–boat isomerization of the six-membered ring, the barrier for this isomerization is relatively high), resulting in a conformational preference at C8 that favors 1,5-HAT occurring at C9. (2) In terms of stereoselectivity, the steric hindrance between the spin center at C14 and the axial methyl group at C10 restricts the rotation around the C13–C14 bond, thereby enabling the diradical to undergo coupling stereoselectively.

As a key late-stage step in this total synthesis, the Norrish–Yang photocyclization exhibits high chemoselectivity and efficiency. It regulates selectivity through C–H bond dissociation energy and restricted bond rotation, constructing a 6/6/4 fused ring system with three contiguous quaternary carbons. Moreover, the reaction can be performed on a 300 mg scale, balancing selectivity, efficiency, and practicality.

#### 1.2 Synthetic study toward phainanoids

**Scheme 4:**
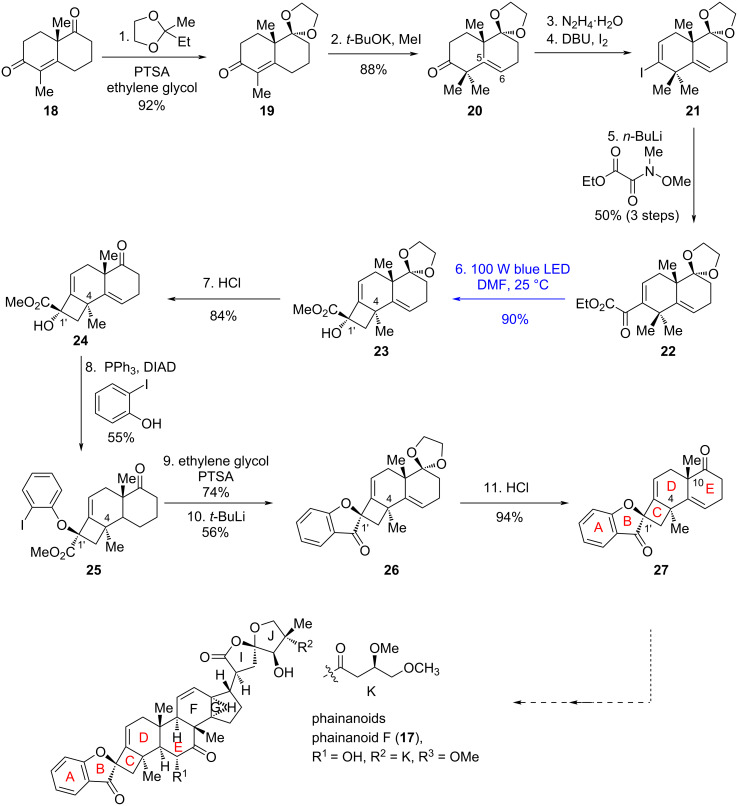
Synthetic study toward phainanoids.

Phainanoids are highly modified triterpenoids first isolated from *Phyllanthus hainanensis* by Yue's group [24–25]. Biologically, these molecules exhibit intriguing immunosuppressive activities, with phainanoid F (**17**) standing out as the most potent: it inhibits the proliferation of T and B lymphocytes with an IC_50_ value of 2.04 ± 0.01 nM. Structurally, phainanoids are characterized by a highly modified dammarane-type triterpenoid core, featuring an unprecedented 4,5-spirocyclic B/C ring system and a [4,3,1]propellane F/G/H ring fragment. In 2024, Yang's group achieved the construction of the ABCDE pentacyclic skeleton of phainanoids, leveraging Norrish–Yang cyclization to accomplish the regio- and stereoselective assembly of the rigid, sterically congested benzofuranone-based 4,5-spirocycle [26]. Starting from Wieland–Miescher ketone (**18**, Scheme 4), the synthesis proceeded through a sequence of transformations: the nonconjugated carbonyl was chemoselectively protected as ketal **19**; the unprotected ketone then underwent α-methylation to provide **20**; and subsequent conversion of the ketone in **20** to the vinyl iodide in **21** – via hydrazone formation, lithium–halogen exchange, and final nucleophilic substitution – secured the Norrish–Yang cyclization precursor **22**. Following systematic optimization of reaction conditions, irradiation of **22** with 100 W blue LEDs at room temperature constructed a single diastereoisomer **23** in 90% yield. From **23**, the ABCDE pentacyclic skeleton of phainanoids (**27**) was ultimately established via a Mitsunobu reaction, intramolecular nucleophilic substitution with in situ-generated aryllithium, and protecting group manipulations.

**Scheme 5:**
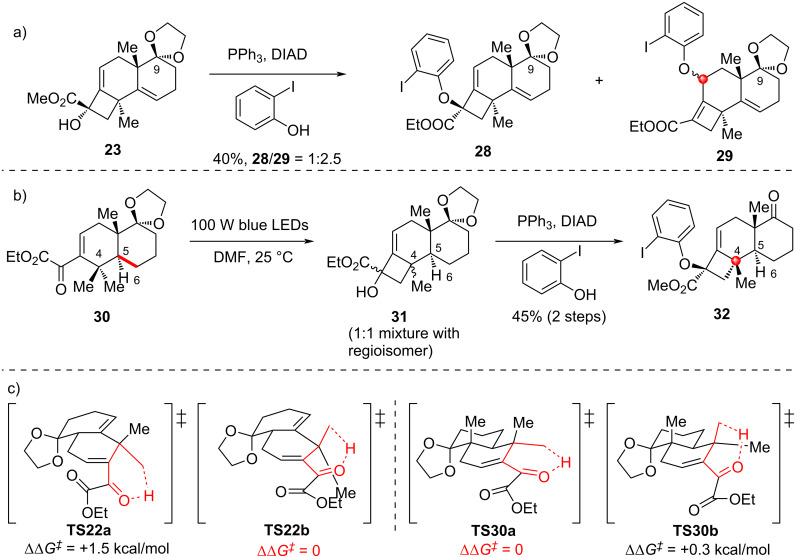
a) Mitsunobu reaction of the C9 ketal; b) Norrish–Yang cyclization of the saturated C5–C6; c) calculated Gibbs free energy difference (ΔΔ*G*‡) for 1,5-HAT processes of **22** and **30**.

Notably, under Mitsunobu conditions, **23** – with its C9 ketal remaining intact – led to **28** and **29** in 40% total yield, with a 1:2.5 ratio favoring the undesired regioisomer **29** (Scheme 5a). These two isomers arise from S_N_2 and S_N_2' mechanisms, respectively. Furthermore, when **30** (bearing a saturated C5–C6 bond) was subjected to Norrish–Yang cyclization conditions (Scheme 5b), the two methyl groups at C4 initially underwent non-selective 1,5-HAT, resulting in a loss of both regio- and stereoselectivity. In both cases, the functional groups on the ring E likely exert a significant influence on the conformation of the substrates, thereby modulating their reactivities. Density functional theory calculations show that **22** prefers to proceed its annulation through **TS22b** (ΔΔ*G*^‡^ = 0) to afford **23** rather than that via **TS22a** (ΔΔ*G*^‡^ = 1.5 kcal/mol). In contrast, annulation of **30** undergoes through the low barrier difference between **TS30b** (ΔΔ*G*^‡^ = 0.3 kcal/mol) and **TS30a** (ΔΔ*G*^‡^ = 0), resulting in the formation of **31** as a 1:1 mixture of regioisomers (Scheme 5c). In this work, to address unsatisfactory C4 stereochemistry in initial synthesis, the authors introduced a double bond to alter substrate conformation, enabling stereochemical inversion of the Norrish–Yang reaction. This achieved efficient construction of benzofuranone-based 4,5-spirocycles with contiguous all-carbon quaternary centers, offering a conformational regulation protocol for stereocontrol in complex polycyclic systems.

#### 1.3 Avarane-type meroterpenoids

**Scheme 6:**
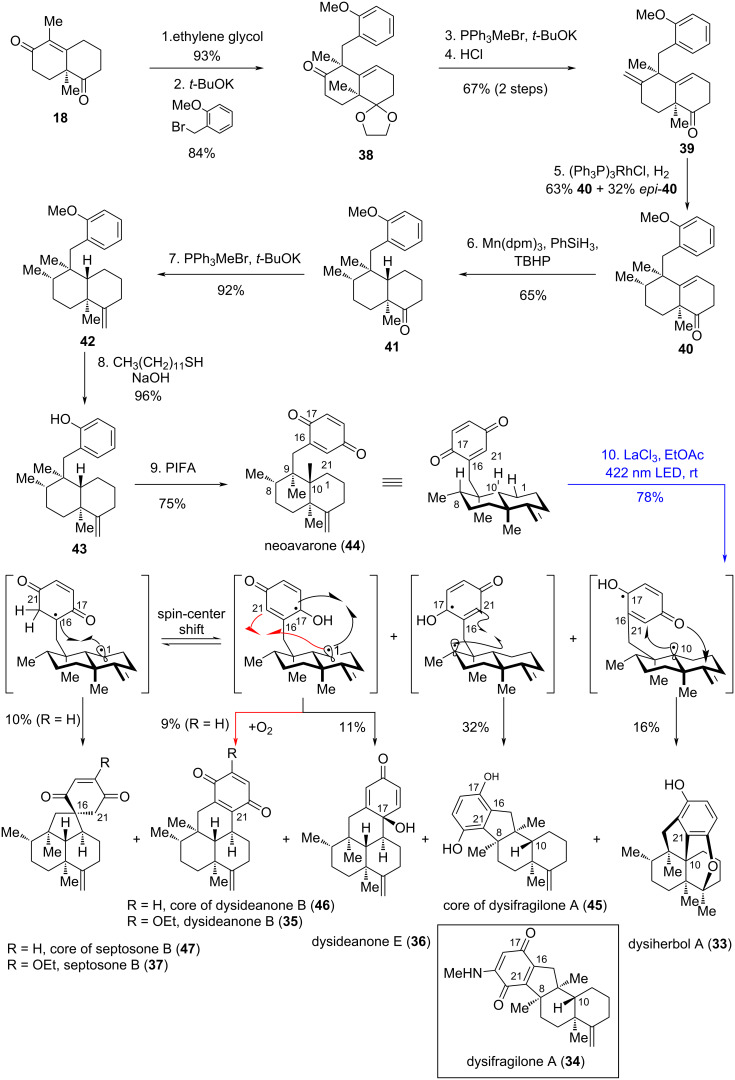
Total synthesis of avarane-type meroterpenoids.

In 2024, Yang's and Zhang's groups employed a quinone-based, acid-promoted Norrish–Yang cyclization to achieve the stereoselective construction of multiple avarane-type meroterpenoids [27], including dysiherbol A (**33**) [28], dysifragilone A (**34**) [29], dysideanone B (**35**) [30], dysideanone E (**36**) [28], and the core structure of septosone B (**37**) [31] (Scheme 6). All these compounds were first isolated from South China Sea sponges of the genus *Dysidea* by Lin's group. Dysiherbol A (**33**) exhibits NF-κB inhibitory activity (IC_50_ = 0.49 μM) and cytotoxicity against the human myeloma cell line NCI H-929 (IC_50_ = 0.58 μM). Dysifragilone A (**34**) displays stronger inhibition of NO production than hydrocortisone, with an IC_50_ of 6.6 μM. Dysideanone B (**35**) shows cytotoxicity against HeLa and HepG2 human cancer cell lines, with IC_50_ values of 7.1 and 9.4 μM, respectively. Septosone B (**37**), featuring an unusual spiro[4.5]decane scaffold, exhibits NF-κB inhibitory activity with an IC_50_ of 27 μM.

Given that **33**–**37** were isolated from the same genus, Yang's group proposed that they might share a common biosynthetic precursor. Leveraging this insight and their knowledge of the Norrish–Yang reaction, they hypothesized that irradiating neoavarone (**44**) would trigger a quinone-based Norrish–Yang cyclization, enabling divergent synthesis of these meroterpenoids (or their core structures). In Yang's divergent total synthesis, Wieland–Miescher ketone (**18**) served as the starting material. Selective acetalization of the non-conjugated ketone in **18**, followed by diastereoselective α-alkylation, produced **38**. A Wittig reaction and subsequent deketalization converted the ketone in **38** to the terminal alkene **39**, allowing for subsequent sequential chemoselective hydrogenations: first, hydrogenation of the *exo*-olefin using Wilkinson’s catalyst proceeded with moderate diastereoselectivity; this was followed by Mn(III)-catalyzed metal-hydride hydrogen atom (MHAT) transfer to reduce the endocyclic olefin, forming **41** as a single diastereomer. Subsequent transformations – including a Wittig reaction, demethylation, and oxidation of the resulting phenol to a *p*-benzoquinone – advanced **41** to neoavarone (**44**). After screening various Lewis acids (including AlCl_3_, Mg(OTf)_2_, Yb(OTf)_3_, etc.), it was finally found that treatment of **44** with irradiation at 422 nm in the presence of the optimal Lewis acid LaCl_3_ furnished the natural products dysiherbol A (**33**) and dysideanone E (**36**), along with the core structures of dysifragilone A (**45**), dysideanone B (**46**), and septosone B (**47**) in the highest total yield of 88% and product divergence. In comparison, the total yield in the absence of any Lewis acid was 66%. Dysifragilone A (**34**) was obtained over four steps from **45**, while dysideanone B (**35**) was completed from **46** via oxidative ethoxylation. The diversity of the key photoreaction stems from three factors: (1) the ability of the excited quinone moiety in **44** to abstract hydrogen atoms from distinct positions; (2) delocalization of the semiquinone radical; (3) the involvement of a spin-center-shift (SCS) process in forming the core of septosone B (**47**) [32–33]. The influence of Lewis acids is partially attributable to two effects: (1) the observed elongation of the lifetime of the singlet excited state (from 0.846 ± 0.015 ns to 0.912 ± 0.014 ns), which reduces undesired photophysical cycles and thereby enhances selectivity; and (2) promotion of subsequent ground-state processes [34–35].

In this work, the authors innovatively applied the Norrish–Yang reaction of *p*-benzoquinone to the synthesis of avarane-type meroterpenoids. Starting from the common precursor quinone, they generated diverse biradical intermediates via regioselective hydrogen transfer (δ-H or ε-H), further constructing various C–C bonds. This enabled efficient synthesis of the core skeletons of five natural products. The divergent strategy significantly reduces redundant synthetic steps, embodies synthetic economy of "one precursor for multiple targets", and provides a valuable scheme for building structurally diverse natural product libraries.

#### 1.4 Gracilisoids A–I

**Scheme 7:**
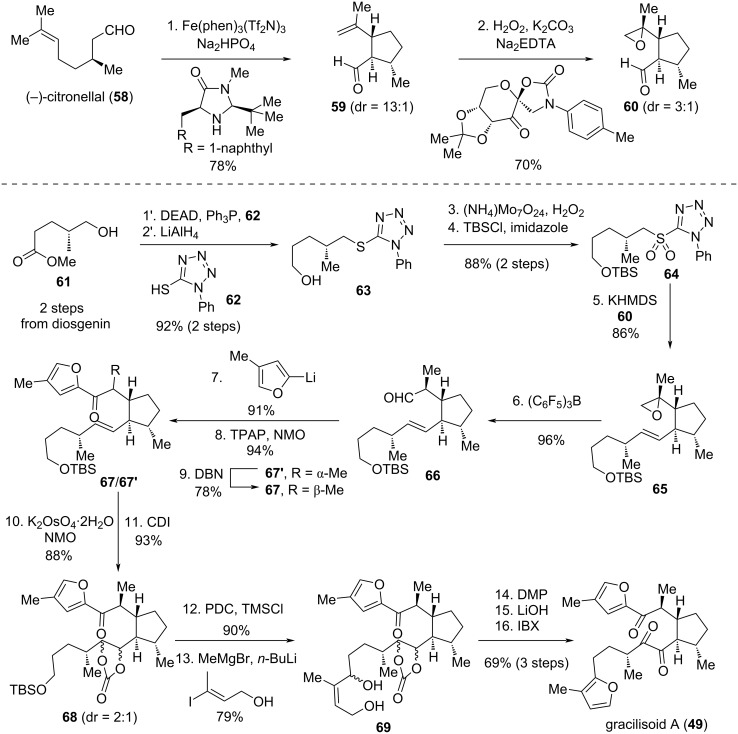
Total synthesis of gracilisoid A.

In 2025, Li's group exemplified the “Isolation-Synthesis-Functionality” research concept for a new class of sesterterpenoids, namely gracilisoids A–I (**49–57**) [36]. Gracilisoids A–E (**49–53**) were isolated from the ethnomedicinal plant *Eurysolen gracilis* of the Lamiaceae, while UPLC-MS/MS analysis of the crude leaf extract indicated trace amounts of gracilisoids F–I (**54**–**57**) in the plant material. Li’s group pursued the divergent total synthesis of **49**–**57** via a bioinspired strategy, incorporating a Norrish–Yang cyclization/α-hydroxyketone rearrangement tandem reaction and late-stage biomimetic photooxidation as key steps. The synthetic endeavour began with the total synthesis of **49**, which served as both the envisioned precursor for Norrish–Yang cyclization and the divergent starting point for accessing the other eight natural products (**50**–**57**). As an initial lead, two commercially available starting materials: (−)-citronellal (**58**) and diosgenin were employed (Scheme 7). (–)-Citronellal (**58**) was first converted to **59** via enantioselective intramolecular aldehyde α-alkylation using MacMillan's protocol, subsequently undergoing Shi's asymmetric epoxidation to give rise to epoxide **60** as a 3:1 mixture of diastereomers. These were not separated until step 8 due to poor separability at this stage. Concurrently, diosgenin was then processed through a known two-step sequence to **61**, followed by Mitsunobu reaction, ester reduction, thioether oxidation, and silylation of the primary alcohol to furnish sulfone **64**. The two key fragments – aldehyde **60** and sulfone **64** – were merged via Julia–Kocienski olefination to construct alkene **65**. Treatment of **65** with (C_6_F_5_)_3_B triggered a Meinwald rearrangement, generating aldehyde **66**. Nucleophilic addition, oxidation of the resulting alcohol, and base-promoted epimerization at C6 of **67**' delivered **67**. Subsequent dihydroxylation of the alkene in **67** and protection of the resulting 1,2-diol as a cyclic carbonate delivered **68**. A one-pot desilylation/oxidation of **67** produced an aldehyde, which was subject to selective nucleophilic addition in the presence of a ketone, allowing for access to the furan precursor **68** [37]. Oxidation–cyclization–aromatization of **68** with Dess–Martin periodinane (DMP) constructed the southern furan moiety of **69**. Finally, hydrolysis of the carbonate followed by oxidation of the resulting diol completed the Norrish–Yang cyclization precursor gracilisoid A (**49**).

**Scheme 8:**
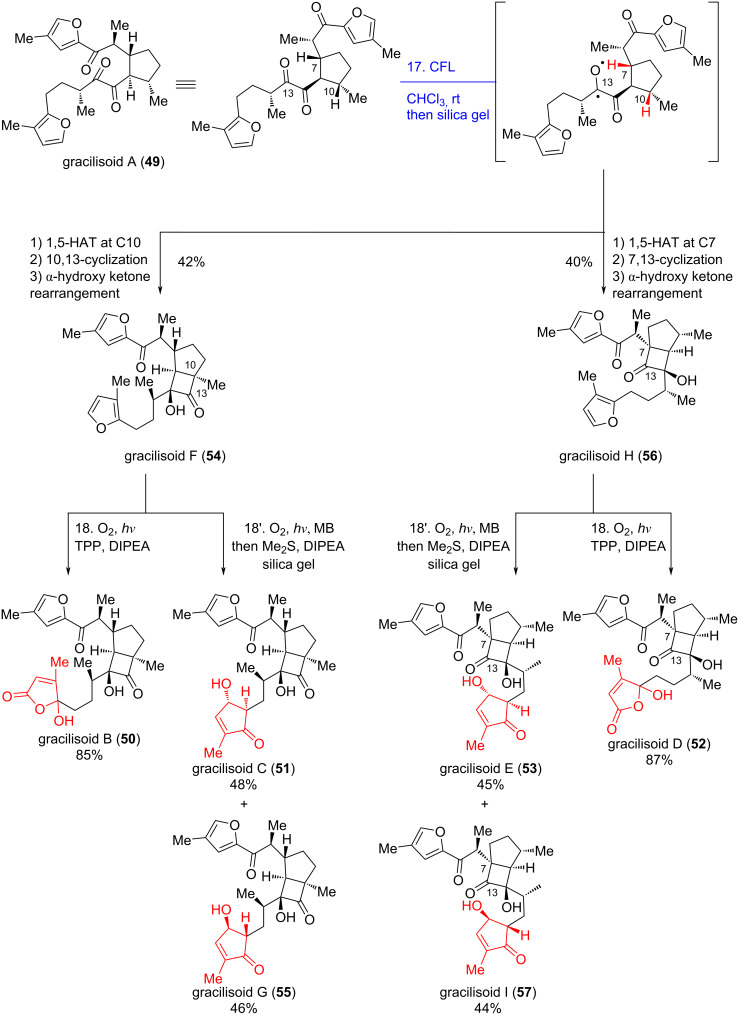
Divergent total synthesis of gracilisoids B–I.

Irradiation of **49** under anaerobic conditions with a CFL, followed by treatment with silica gel, successfully generated a pair of separable regioisomers: gracilisoid F (**54**) in 42% yield and gracilisoid H (**56**) in 40% yield, respectively (Scheme 8). Here, light triggered a regiodivergent Norrish–Yang cyclization, while the acidic nature of silica gel sufficiently promoted an α-hydroxy ketone rearrangement. This type of rearrangement was also observed in Yang’s total synthesis of (+)-cyclobutastellettolide B (**13**) as described above [21]. The driving force for the rearrangement is proposed to stem from 1,2-strain between the substituents at C13/C10 and C13/C7, consistent with observations from Yang's work [21].

**Scheme 9:**
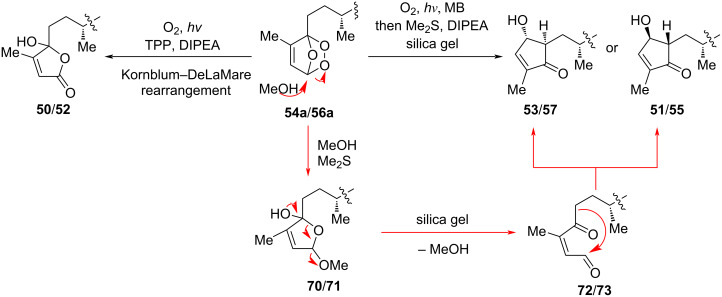
Mechanism of the late-stage biomimetic photooxidation.

Next, peroxides **54a** and **56a** (Scheme 9) – formed via [4 + 2] cycloaddition of singlet oxygen with the southern furan moiety in gracilisoid F (**54**) and gracilisoid H (**56**), respectively – served as branching points in the downstream divergent synthesis. Kornblum–DeLaMare-type rearrangement of **54a** and **56a** assembled gracilisoids B (**50**) and D (**52**), respectively. Alternatively, nucleophilic addition of MeOH to **54a** and **56a**, followed by ring-opening elimination, arrived at the corresponding γ-keto aldehyde intermediates **72**/**73**. Subsequent intramolecular aldol cyclization of these intermediates furnished gracilisoids C/G (**51**/**55**) and E/I (**53**/**57**), respectively.

In this work, inspired by the biosynthetic pathway of natural products, the authors employed a tandem Norrish–Yang photocyclization and α-hydroxy ketone rearrangement as the key step to construct the unique bicyclo[3.2.0]heptane skeleton

of gracilisoids. This strategy ingeniously leverages the high selectivity of photochemical reactions and the strain-release property of rearrangement reactions, enabling the precise construction of multiple stereocenters in one step and overcoming the synthetic challenges posed by contiguous quaternary carbon centers and sterically congested structures in complex bicyclic systems.

### 2 Alkaloids

#### 2.1 Lycoplatyrine A

**Scheme 10:**
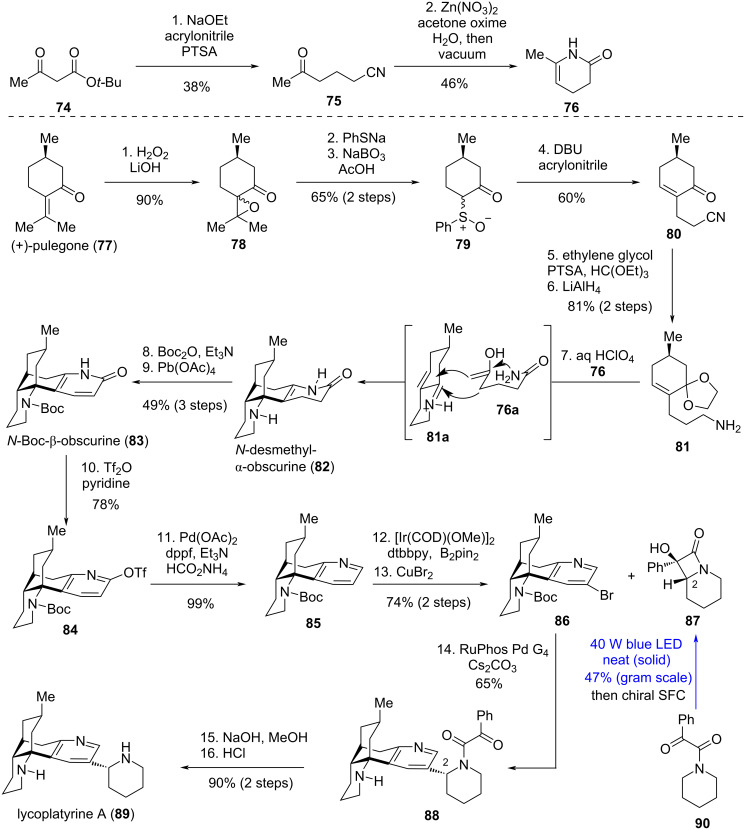
Asymmetric total synthesis of lycoplatyrine A.

In 2020, Sarpong's group disclosed their progress toward a robust and broadly applicable strategy for the C–H functionalization of piperidines and related saturated *N*-heterocycles. This approach involves lactamization via Norrish–Yang cyclization, followed by a strain-release Pd-catalyzed C–C cleavage/cross-coupling protocol [9,11]; the strategy was subsequently applied to the total synthesis of lycoplatyrine A (**89**) in 2021 [38]. Isolated by Low’s group [39], lycoplatyrine A (**89**) belongs to the lycodine-type *Lycopodium* alkaloids – a structurally diverse class of natural products found in plants of the widely distributed genus *Lycopodium*. Guided by biosynthetic hypotheses, Sarpong's group envisioned a divergent total synthesis of five lycodine-type *Lycopodium* alkaloids (including lycoplatyrine A (**89**)) used *N*-Boc-β-obscurine (**83**) as a late-stage common intermediate. Compound **83** was readily accessible via previously reported protocols [40], which featured a diastereoselective formal [3 + 3] cycloaddition between **76** and **81** as the key step. This cycloaddition constructs the three contiguous stereocenters and two C–C bonds of **83** (Scheme 10). Building block **76** was first obtained in 18% overall yield from β-ketoester **74** through a sequence involving Michael addition, decarboxylation, nitrile hydration, and cyclization under vacuum. Aminoketal **81** – a direct precursor to the oxygen-sensitive α,β-unsaturated iminium **81a** – was prepared from (+)-pulegone (**77**) through a six-step manipulation involving epoxidation, epoxide opening with sodium thiophenolate and subsequent concomitant retro-aldol, sulfoxidation, a one pot α-alkylation with acrylonitrile proceeding to thermal *syn*-elimination of phenylsulfenic acid, ketone protection as ketal and nitrile reduction. The two building blocks (**76** and **81**) were then merged through the desired formal [3 + 3]-cycloaddition to generate *N*-desmethyl-α-obscurine (**82**), presumably via in situ-generated intermediates **76a** and **81a**, respectively. Subsequent Boc protection of the cyclic amine in **82**, followed by dehydrogenation, delivered compound **83** in 49% yield over three steps. Pyridone **83** could be funneled into pyridine **85** through *O*-triflation followed by Pd-catalyzed reductive detriflation. Ir-catalyzed *meta*-selective C–H borylation of **85**, followed by bromodeborylation of the pyridine ring, constructing **86 –** setting the stage for the group’s key reaction, in which α-hydroxy-β-lactams (e.g., **87**) serve as surrogates for α-metalated *N*-heterocycles in Pd-catalyzed coupling with aryl halides. Both enantiomers of **87** (only one shown in Scheme 10) participated in a Pd-catalyzed stereoretentive coupling at C2 with aryl bromide **86**, yielding **88** as a single diastereomer. Manipulation of the protecting group of **88** arrived at lycoplatyrine A (**89**). Notably, **87** and related α-hydroxy-β-lactams were obtained via Norrish–Yang cyclization of the corresponding neat α-keto amides in the solid state under blue LED irradiation. Solid-state reactions differ from solution-phase processes due to restraints on molecular motions imposed by the crystal lattice, thereby avoiding side reactions caused by unrestricted molecular motions in solution [41]. It has also been demonstrated that for crystalline 1,2-diketones, the rigid lattice structure locks molecules into a specific conformation, limiting access to certain γ-hydrogens for abstraction and thus enhancing regioselectivity [42]. Enantiopure **87** was obtained by preparative chiral supercritical fluid chromatography (SFC) resolution of the racemate, while racemic **87** was synthesized on a gram scale in 47% yield from **90** via a Norrish–Yang reaction.

**Scheme 11:**
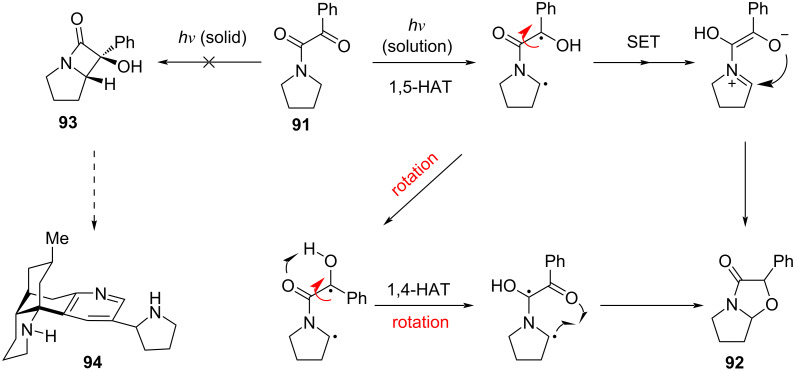
Photoreaction of pyrrolidine-derived phenyl keto amide.

However, under the established photochemical conditions for generating **87**, the corresponding pyrrolidine-derived species **93** is not formed (Scheme 11). In contrast, solution-phase irradiation of the same pyrrolidine-derived phenyl keto amide substrate **91** with blue LEDs produces the pyrrolidine-fused 4-oxazolidinone (*N*,*O*-acetal) **92**, precluding preparation of the pyrrolidine analog of lycoplatyrine A (**94**) by this method. Compound **92** is presumably formed via either the radical mechanism [41] or possibly undergoes SET to form a zwitterion as an intermediate step prior to cyclization [35,43–44]. It is noted that such molecular motions (rotations) are possible in solution but prevented by crystal-lattice restraints in the solid state [37].

In this synthesis, Sarpong et al. utilized the Norrish–Yang reaction to construct a β-lactam, an α-metallated piperidine equivalent, overcoming poor yields and stereoselectivity in traditional methods. Its palladium-catalyzed cross-coupling with 2-bromolycodine via β-lactam C–C cleavage enabled stereoretentive coupling, efficiently synthesizing lycoplatyrine A and its epimers. This strategy merges photochemical selectivity with cross-coupling efficiency, avoiding reactive metal incompatibility and enabling precise stereocontrol for chiral nitrogen heterocycles.

### 3 Antibiotics

#### 3.1 Synthetic study toward γ-rubromycin

**Scheme 12:**
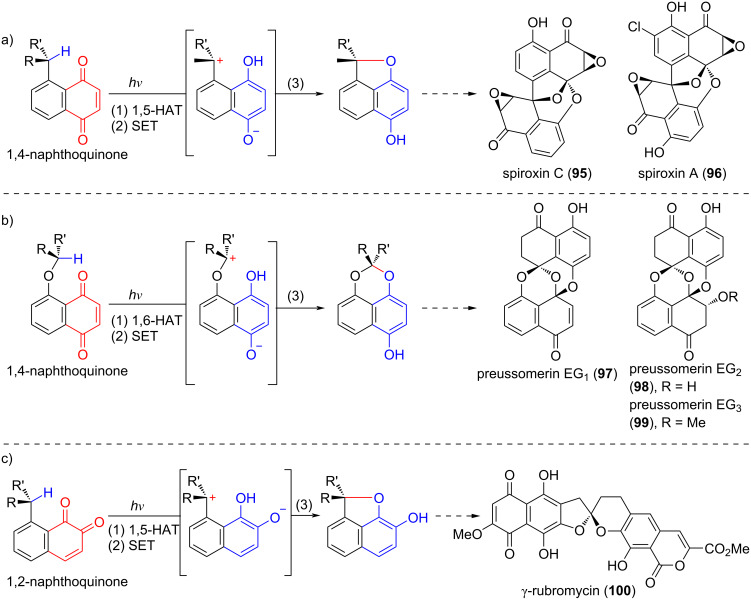
Photoredox reactions of naphthoquinones.

Since 2017, Suzuki's group has developed a stereoretentive photoredox reaction of naphthoquinones initiated by Norrish type-II hydrogen abstraction [45–51]. The mechanism involves three consecutive photoinduced events: (1) hydrogen abstraction by the excited quinone to generate a biradical species; (2) intramolecular SET process converting the biradical to the corresponding zwitterion; (3) oxy-cyclization to afford the product. This process constitutes an intramolecular redox reaction, wherein the quinone is reduced to a hydroquinone while the proximal C–H bond is oxidized to a C–O bond. Although related photoredox reactions had precedents [9], no systematic studies on their scope and limitations had been reported. Suzuki's group optimized the reaction conditions and thoroughly explored the substrate scope, encompassing 1,5-HAT and 1,6-HAT of 1,4-naphthoquinones as well as 1,5-HAT of 1,2-naphthoquinones (Scheme 12). Ultimately, this approach enabled the total synthesis of spiroxin C (**95**) [45], spiroxin A (**96**) [48], preussomerin EG_1_ (**97**), preussomerin EG_2_ (**98**), and preussomerin EG_3_ (**99**) [49], alongside model studies on γ-rubromycin (**100**) [50–51].

**Scheme 13:**
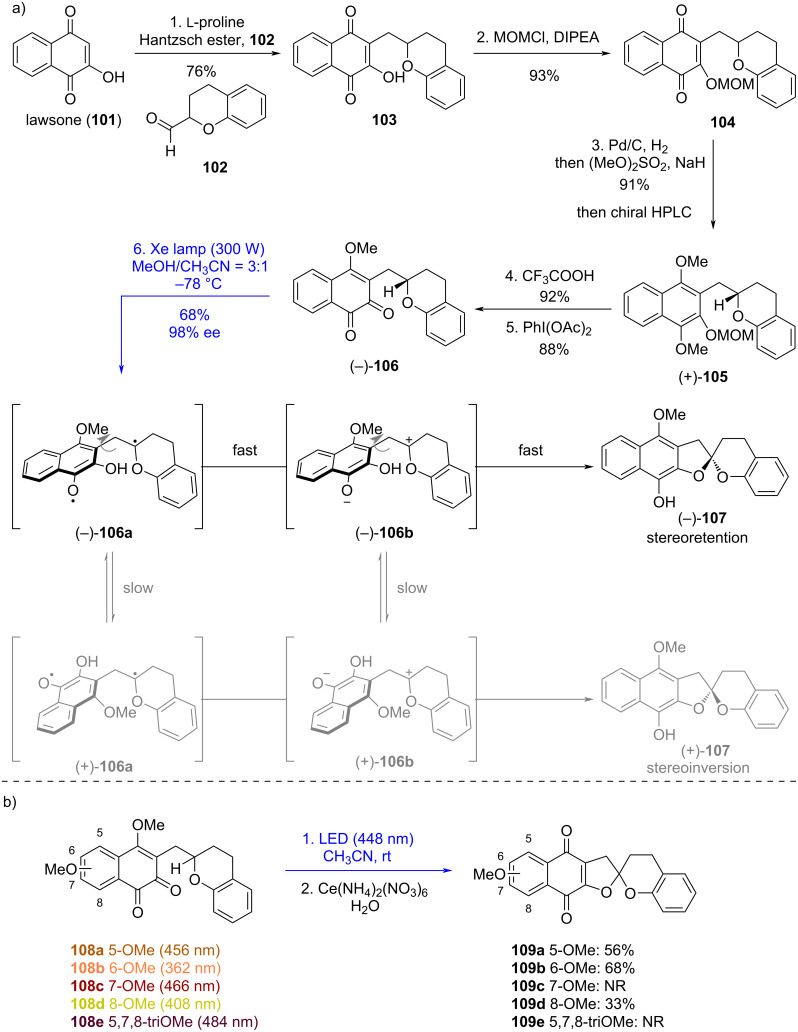
Synthetic study toward γ-rubromycin.

γ-Rubromycin (**100**) is a prominent member of the rubromycin family of natural products, characterized by a [5,6]-bisbenzannulated spiroketal moiety as its central structural motif. This key feature is critical to its potent biological activities, including strong inhibition of human telomerase [52], as well as its established roles as an effective antibiotic and HIV-1 reverse transcriptase inhibitor [53–54]. In 2018, Suzuki’s group employed their developed photoredox reaction to carry out a model study on the chiral [5,6]-spiroketal core of γ-rubromycin [50]. 1,2-Naphthoquinone **106** was selected as the model substrate for the photoredox reaction (Scheme 13a). Lawsone (**101**) underwent reductive alkylation with aldehyde **102**, producing **103**, which was then protected as its OMOM ether to yield **104**. One-pot hydrogenation of quinone **104** afforded the corresponding hydroquinone, which, upon subsequent methylation, furnished **105**. Removal of the MOM group from **105** followed by oxidation of the resulting phenol delivered 1,2-naphthoquinone **106**. Notably, enantiomer (−)-**106** could be prepared from (+)-**105**, which was in turn obtained by preparative HPLC on a chiral stationary phase from (±)-**105**. Following extensive screening, it was found that photoirradiation of quinone (−)-**106** under the optimized conditions (300 W Xe lamp, MeOH/CH_3_CN = 3:1, −78 °C) induced the formation of the five-membered ring, forming spiroacetal (−)-**107** in 68% yield with nearly complete configurational integrity (98% ee). This high stereoselectivity is attributed to three factors: (1) polar solvents enhance yields by accelerating electron transfer for zwitterionic intermediate (e.g., (−)-**106b** or (+)-1**06b**) formation; (2) protic solvents (e.g., MeOH) improve ee values through H-bonding stabilization, which reduces intermediate flexibility; (3) low temperatures enhance the optical purity by slowing conformational changes without compromising the reaction rate. These rationales for stereoretention are equally applicable to photoredox reactions of other substrates (vide infra), and therefore will be omitted there.

In 2024, Suzuki’s group further explored the feasibility of their photoredox strategy for constructing the γ-rubromycin spiroacetal core using multi-functionalized 1,2-naphthoquinone substrates [51]. This study focused on substituent effects of methoxy groups at different positions (C5–C8) on the naphthoquinone chromophore (Scheme 13b). Key findings revealed that substrates bearing methoxy groups at C5, C6, or C8 (**108a**, **108b**, **108d**) underwent the desired photochemical reaction (Scheme 13b), yielding spiroacetals **109a**, **109b**, and **109d** following CAN (cerium(IV) ammonium nitrate) oxidation. In contrast, C7-methoxy-substituted analogs (**108c**, **108e**) remained unreactive. Reactivity correlated with UV–vis absorption profiles: reactive substrates exhibited λ_max_ < 460 nm (yellow-orange), whereas unreactive ones showed λ_max_ > 460 nm (dark red-purple). This suggests insufficient excitation energy for Norrish type-II hydrogen abstraction in the latter. Such chromophore-dependent reactivity provides critical insights for designing viable intermediates in the total synthesis of γ-rubromycin, emphasizing the need to avoid C7-oxygenation at early stages.

#### 3.2 Preussomerins

**Scheme 14:**
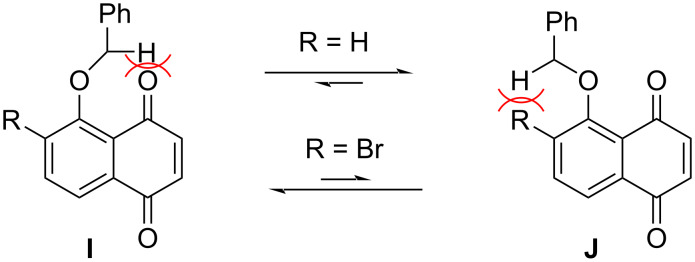
Substituent-dependent conformational preferences.

Preussomerins EG_1_ (**97**), EG_2_ (**98**), and EG_3_ (**99**) – isolated from the endophytic fungus *Edenia gomezpompae* in *Callicarpa acuminata* – exhibit strong antifungal activity against phytopathogens such as *Phytophthora* and *Fusarium*. Among these, preussomerin EG_1_ (**97**) is the most potent, with IC_50_ values of 57.71 × 10^−5^ M against *Phytophthora parasitica*, 5.61 × 10^−5^ M against *Phytophthora capsici*, and 1.45 × 10^−5^ M against *Fusarium oxysporum*, while showing no significant inhibition (>5.75 × 10^−4^ M) against *Alternaria solani* [55]. In 2023, Suzuki's group pioneered the enantioselective total syntheses of preussomerins **97**–**99** through their photoredox strategy [49]. This study addressed the key challenge of controlling spiroacetal stereoselectivity through a 1,6-HAT process – a less favorable pathway compared to the previously employed 1,5-HAT. Initial feasibility studies revealed that the non-brominated substrate exhibited poor reactivity in the photochemical 1,6-HAT reaction (Scheme 14), as its unreactive conformer **J** dominated the equilibrium. In contrast, the brominated analogue achieved high efficiency: the bulky bromine atom sterically biased the population toward the reactive conformer **I**, where the benzylic C–H bond is optimally positioned for 1,6-HAT.

**Scheme 15:**
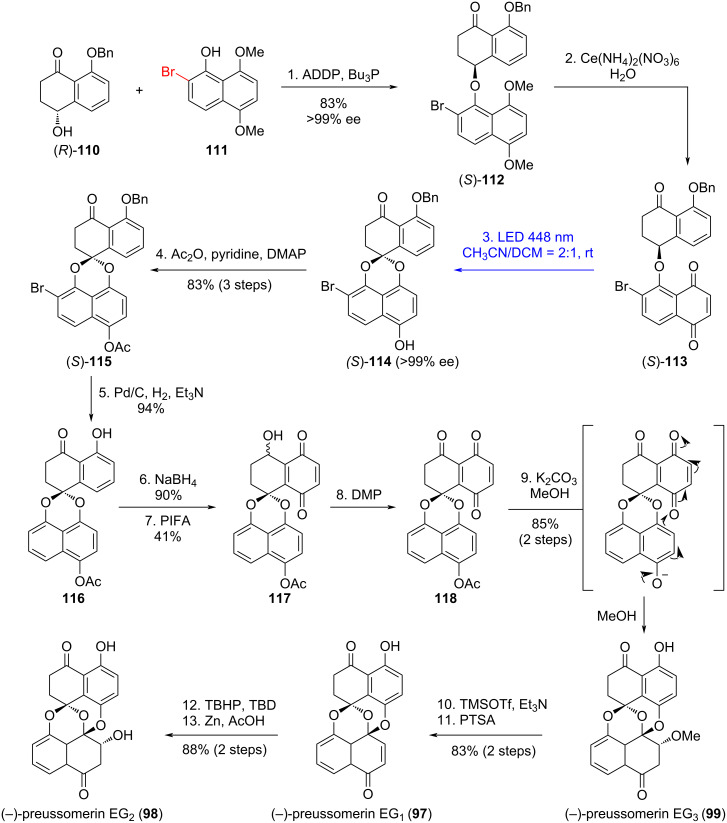
Total synthesis of preussomerins EG_1_, EG_2_, and EG_3_.

Therefore, (*S*)-**113** was selected as the precursor for the key photoredox reaction (Scheme 15). To prepare this, a Mitsunobu reaction between two known compounds – (*R*)-**110** and **111** – afforded (*S*)-**112** (>99% ee after recrystallization), which was then oxidized to yield the key naphthoquinone (*S*)-**113**. As expected, the photoredox reaction of (*S*)-**113** proceeded efficiently under blue LED irradiation (448 nm) in CH_3_CN/CH_2_Cl_2_ at room temperature, delivering spiroacetal (*S*)-**114** with complete retention of configuration (>99% ee). The subsequent one-pot acetylation generated (*S*)-**115** in 83% overall yield over three steps from (*S*)-**112**. The following functional group manipulations of (*S*)-**115** – including debenzylation/debromination, ketone reduction, hydroquinone oxidation, and alcohol oxidation – led to the labile ketone **118**. Completion of preussomerin EG_3_ (**99**) from **118** relied on a simple sequence including a formal intramolecular 1,6-addition, followed by simultaneous methanol trapping of the resulting electrophilic quinone. Notably, **116** resisted oxidation to **118**, presumably due to carbonyl-induced π-electron depletion, whereas compound **118** proved resistant to direct conversion into preussomerin EG_3_ (**99**) under various conditions. Alternatively, preussomerin EG_1_ (**97**) was obtained in 83% yield over two steps from preussomerin EG_3_ (**99**) via TMSOTf/Et_3_N-mediated β-elimination of methanol followed by desilylation. Finally, stereoselective epoxidation and reductive opening of the oxirane ring enabled the total synthesis of preussomerin EG_2_ (**98**) from preussomerin EG_1_ (**97**) in 88% yield over two steps.

In this synthesis, the stereospecific intramolecular photoredox reaction of naphthoquinones overcame the limitation of poor stereoselectivity in traditional photochemical reactions, representing the first application of naphthoquinone photoredox processes to the stereospecific construction of complex spirocyclic structures. Its ingenuity lies in utilizing the inherent conformational constraints of the substrate instead of external chiral catalysts, which not only simplifies the reaction system but also solves the challenge of precisely controlling contiguous chiral centers in polycyclic systems.

## Conclusion

This review systematically summarizes the latest advancements in the Norrish–Yang cyclization and related photoredox reactions of dicarbonyls, with a focus on their applications in the total synthesis of bioactive natural products. It highlights the distinct photochemical behaviors of various dicarbonyls, including 1,2-diketones, α-keto esters, α-keto amides, 1,4-quinones, and 1,2-quinones, emphasizing their unique roles in constructing diverse substructures. However, the reaction faces challenges in terms of regioselectivity and stereoselectivity, which are mainly attributed to the influence of substrate structure, substituent effects, and steric hindrance. Meanwhile, for multi-functional group substrates, the presence of potential competitive hydrogen transfer sites tends to lead to the formation of non-target cyclized products, further reducing the reaction specificity. Dicarbonyls are excellent substrates for Norrish–Yang cyclization and related photoredox reactions, owing to their ability to undergo selective activation under mild conditions (e.g., long-wavelength irradiation) – a feature that minimizes competing fragmentation pathways. Notably, different dicarbonyl substrates exhibit distinct reactivity patterns, enabling the construction of diverse architectures including sterically hindered cyclobutanes, spiroketals, and fused polycycles. However, the potential of dicarbonyls in photoreactions warrants further exploration. Future research could investigate a broader range of dicarbonyl substrates to uncover new reactivity modes and expand the structural diversity of accessible natural product scaffolds. Additionally, the highly reactive strained four-membered rings generated from these reactions present exciting opportunities for further functionalization. Studying their downstream transformations – particularly under catalytic conditions – may lead to novel strategies for C–H functionalization and fragment coupling, ultimately advancing the field of target-oriented synthesis. Combining Norrish–Yang reaction with flow chemistry technology to realize the continuous operation of the reaction can improve the safety and controllability of the reaction, and at the same time, it is also conducive to the rapid optimization of reaction conditions and scale-up production. Finally, the Norrish–Yang reaction can utilize artificial intelligence to assist in designing reaction routes in the future, and through big data analysis and machine learning algorithms to quickly screen and optimize reaction conditions, substrate structures, and predict reaction results, it will accelerate the development of new Norrish–Yang reaction systems in the total synthesis of complex bioactive natural products.

## Data Availability

Data sharing is not applicable as no new data was generated or analyzed in this study.
